# Synergy between time-restricted feeding and time-restricted running is necessary to shift the muscle clock in male wistar rats

**DOI:** 10.1016/j.nbscr.2024.100106

**Published:** 2024-09-19

**Authors:** Ayano Shiba, Paul de Goede, Roberta Tandari, Ewout Foppen, Nikita L. Korpel, Tom V. Coopmans, Tom P. Hellings, Merel W. Jansen, Annelou Ruitenberg, Wayne I.G.R. Ritsema, Chun-Xia Yi, Joram D. Mul, Dirk Jan Stenvers, Andries Kalsbeek

**Affiliations:** a Netherlands Institute for Neuroscience, An Institute of the Royal Netherlands Academy of Arts and Sciences (KNAW), Meibergdreef 47, 1105BA, Amsterdam, the Netherlands; b Amsterdam UMC, University of Amsterdam, Laboratory of Endocrinology, Department of Laboratory Medicine, Meibergdreef 9, Amsterdam, the Netherlands; c Amsterdam Gastroenterology, Endocrinology and Metabolism (AGEM), Amsterdam, the Netherlands; d Department of Endocrinology and Metabolism, Amsterdam UMC, University of Amsterdam, Meibergdreef 9, 1105BA, Amsterdam, the Netherlands; e Brain Plasticity Group, Swammerdam Institute for Life Sciences, Faculty of Science, Science Park 904, 1098XH, Amsterdam, the Netherlands; f Centre for Urban Mental Health, University of Amsterdam, Amsterdam, the Netherlands

**Keywords:** Circadian misalignment, Energy metabolism, Wheel running, Time restriction, Liver, Muscle

## Abstract

Circadian disruption is an important factor driving the current-day high prevalence of obesity and type-2 diabetes. While the impact of incorrect timing of caloric intake on circadian disruption is widely acknowlegded, the contribution of incorrect timing of physical activity remains relatively understudied. Here, we modeled the incorrect timing of physical activity in nightshift workers in male Wistar rats, by restricting running wheel access to the innate inactive (light) phase (LR). Controls included no wheel access (NR); access only during the innate active (dark) period (DR); or unrestricted (*ad libitum*) access (ALR). LR did not shift the phase of the muscle or liver clock, but dampened the muscle clock amplitude. As our previous study demonstrated that light-phase restricted feeding did shift the liver clock, but made the muscle clock arrhythmic, we next combined the time restriction of wheel and food access to either the light phase (LRLF) or dark phase (DRDF). LRLF produced a ∼12 h shift in the majority of clock gene rhythms in both skeletal muscle and liver. On the other hand, DRDF was most effective in reducing body weight and the accumulation of fat mass. Therefore, in order to shift the muscle clock in male Wistar rats, synergy between the timing of feeding and physical activity is necessary. These findings may contribute to further improve the design of lifestyle strategies that try to limit metabolic misalignment caused by circadian disruption.

## Highlights

• Voluntary wheel running (VWR) in the active phase boosts the muscle clock amplitude.
• VWR in the active phase blunts weight gain most effectively.
• VWR in the inactive phase dampens the skeletal muscle clock gene expression rhythm.
• Inactive-phase restricted VWR and feeding shifts the liver and muscle clock by ∼12 h.
• Active-phase restricted VWR and feeding prevents weight gain and adiposity the best.

## Graphical abstract

**Figure:**
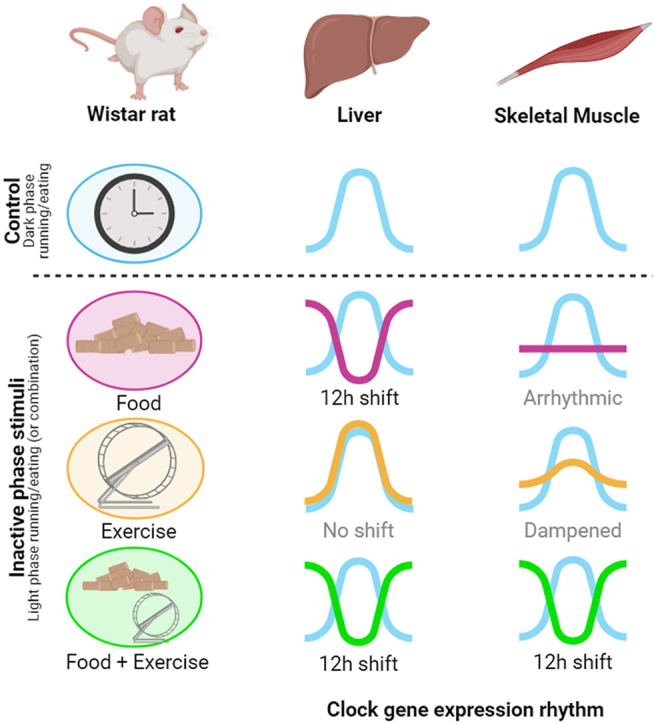

## 1 Introduction

The circadian timing system and physical locomotor activity are closely intertwined. Indeed, physical activity in a running wheel has long been used as the primary quantitative model of circadian behavior (Siepka and Takahashi, 2005). Disturbance of the circadian clock strongly affects daily physical activity patterns and *vice versa*. Lesioning the master pacemaker of the circadian timing system in the suprachiasmatic nucleus (SCN), as well as whole-body knockout of several core clock genes results in arrhythmic running wheel activity, even under regular light/dark conditions (Cermakian et al., 2001; Eastman et al., 1984). On the other hand, physical activity is also one of the best-known non-photic Zeitgebers that can promote a phase-shift of the central SCN clock (Dallmann and Mrosovsky, 2006).

Physical activity is also a potent Zeitgeber for peripheral clocks, especially for skeletal muscle, with several lines of evidence indicating that physical activity can modulate local peripheral clocks (Kemler et al., 2020; Schroder and Esser, 2013). For example, 2 h of either voluntary or forced running during the inactive (light) period for four weeks shifted the peripheral clocks in skeletal muscle and lungs, but not the central clock in mice (Wolff and Esser, 2012). In contrast, four weeks of free access to a running wheel (which results in the vast majority (>95%) of running during the active dark phase) resulted in a phase-advance of the rhythms of *Per1, Per2, Reverbα*, and *Dbp* in the liver and white adipose tissue, but not in brown adipose tissue and skeletal muscle, compared to sedentary control mice (Yasumoto et al., 2015). Peak expression levels of *Per1*, *Per2,* and *Reverbα* in the skeletal muscle were increased in the running mice, although their acrophase did not differ (Yasumoto et al., 2015). Finally, running restricted to 4 h at the beginning or end of the active dark period delayed the expression peak of *Per2* in liver and kidney, but not in heart or SCN (Schroeder et al., 2012). Taken altogether, these findings indicate that the timing of (voluntary) running has differential impact on several peripheral clocks. In addition, the shift of clock gene rhythms is larger with forced running than voluntary running (Sasaki et al., 2016), which highlights the importance of the experimental setup. It is important to note that the majority of wheel running studies including the ones above were all conducted in C57BL/6 *Per2::Luc* mice (Sasaki et al., 2016; Schroder and Esser, 2013; Schroeder et al., 2012; Wolff and Esser, 2012).

Since the pioneering work by Damiola and colleagues (Damiola et al., 2000), it has been acknowledged that eating restricted to the inactive light period causes a 12 h shift in the liver clock. We expanded on these findings by demonstrating that also in male Wistar rats feeding restricted to the light-phase promoted anti-phasic clock gene expression rhythms in the liver, but also dampened the muscle clock rhythm (de Goede et al., 2018a; Oosterman et al., 2020). This arrhythmicity of the muscle clock was likely not attributable to the shift in feeding behavior, but changes in physical activity rhythms (De Goede et al., 2020; de Goede et al., 2018a, de Goede et al., 2018b; Oosterman et al., 2020; Opperhuizen et al., 2016). These differential effects on the liver and muscle clock, i.e. circadian misalignment, potentially mediate the negative metabolic outcomes observed in rats that could only eat during the light-phase (de Goede et al., 2019) or in people performing shift work (Depner et al., 2014). Therefore, we investigated whether time-restricted running or the combination of time-restricted running and time-restricted feeding could be used to shift the muscle clock and align the liver and muscle clock again (i.e. repair or prevent the peripheral misalignment).

## 2 Materials and methods

### 2.1 Ethics approval statement

All experimental procedures were performed in accordance with the European guidelines for laboratory animals (EU directive 2010\63\EU) and approved by the Dutch Central Committee for Animal Experiments (CCD permits: AVD801002016693, AVD8010020172424, and AVD80100202216157) and the Agency for Animal Welfare (IvD) committee of the Netherlands Institute of Neuroscience (Royal Dutch Academy of Sciences).

### 2.2 Animals and housing

#### 2.2.1 Experiment-1: running restricted to light or dark phase

A total of 199 male Wistar rats (WU, Charles River) were used for this study, weighing 240–280 g (around 8 weeks old) upon arrival in batches of 12–20 rats. After 1 week of acclimatization, rats were individually housed in a temperature- (21–23 °C), humidity- (40–60%) and light-controlled room [12:12 h light/dark cycle; 280 (±80) Lux (light phase):<5 Lux (dark phase); with lights on at Zeitgeber time (ZT) 0 and lights off at ZT12] within the animal facility of the Netherlands Institute of Neuroscience. The maximum light intensity was observed in the front area of each cage, while the minimum intensity was observed in the back side of each cage next to the running wheel. Rats were housed in custom-made cages [522 (w) × 582 (l) × 412 (h) mm], in which they could freely move between the home cage compartment and a vertical 36 cm-diameter stainless-steel running wheel (Model 80850MS, Campden Instruments). Pelleted chow (#2918, Teklad Irradiated Global 18% Protein Rodent Diet, Envigo) and a bottle with tap water were available *ad libitum* in the home cage throughout the entire experiment. The running wheel could be blocked using an Arduino controlled actuator. During the braking procedure, the actuator gradually increased the friction on the running wheel during 20 s until a full brake was accomplished so that the rats would not be injured while running during the braking procedure.

#### 2.2.2 Experiment 2: running and eating restricted to light or dark phase

A total of 71 male Wistar rats (WU, Charles River) were used for this study, weighing 240–300 g upon arrival in batches of 35/36 rats. After 1 week of acclimatization, rats were pair-housed in the same experimental condition as Experiment-1.

### 2.3 Experimental design

#### 2.3.1 Experimental cohorts

##### 2.3.1.1 Experiment-1: running restricted to light or dark phase

**Fig. 1:**
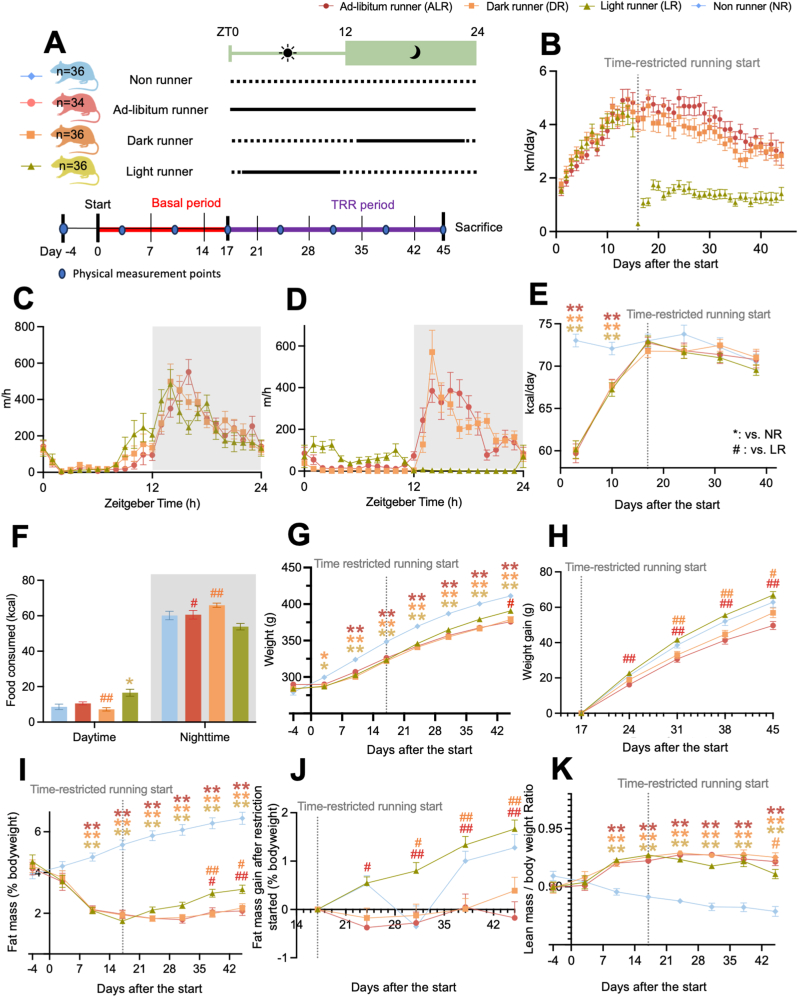
Voluntary running wheel activity restricted to the light phase has less beneficial physical outcomes than running wheel activity restricted to the dark phase. **A:** Experimental design with the timeline of the experiment, colour coding of each experimental group and the sample size. Solid black line = unlocked wheel phase, dotted line = locked wheel phase. **B:** The average daily running distance of each group during the experiment. **C-D:** Daily running pattern during the baseline (C) and time-restricted phase (D) Ad-libitum (ALR, in red, n = 19), Dark (DR, in orange, n = 19), Light (LR, in yellow green, n = 17). **E:** Daily food intake in kcal. **F:** Day and night food intake during the final week. Non-runner (NR, in blue) n = 11; ALR, n = 14; LR, n = 17; DR, n = 17. **G:** Body weight development. **H:** Gain of body weight from the start of the time-restricted running period. **I:** Fat mass in percentage per body weight. **J:** Gain of body fat from the start of the time-restricted running period. **K:** Lean mass in percentage per body weight. The start of the time-restricted running period is represented by a vertical grey dotted line. The grey shaded area (in C, D and F) represents the dark (inactive) phase. Data are presented as the mean ± SEM. Significant difference from NR (∗) or from LR (#) compared to the groups of color code. ∗ or #: P < 0.05, ∗∗ or ##: P < 0.01 by two-way ANOVA followed by Tukey HSD *post-hoc* test. (For interpretation of the references to color in this figure legend, the reader is referred to the Web version of this article.)

After one week of acclimatization, rats were randomly assigned to one of four experimental groups: no access to running wheel (NR); unrestricted (or *ad libitum*) access to a running wheel (ALR); access to the running wheel restricted to the dark period (ZT13-23; DR); and access to the running wheel restricted to the light period (ZT1-11; LR) (Fig. 1A). Body weight was measured weekly. Fat mass and lean mass (*i.e.* muscle, bone and organ mass) were also measured weekly using an EchoMRI-500 QMR system (EchoMRI, Houston, TX, USA). Food intake was measured between ZT1-2 and ZT10-11, 2–3 times weekly to estimate the light-phase and dark-phase food intake. The average value was considered as the weekly food intake. During the basal running period (17 ± 2 days), all ALR, DR, and LR rats had unrestricted access to running wheels to get accustomed to them. After the basal period animals with a running wheel were randomized, using RandoMice (van Eenige et al., 2020), over the 3 experimental running groups based upon body weigt, fat percentage and running activity. During the restricted running period (28–29 days), access to the wheels was restricted to a 10-h time period in the dark (ZT13-23 for DR group) or light period (ZT1-11 for LR group), while the ALR group continued with unrestricted access to wheels. The NR control group was housed without a running wheel throughout the experiment. After the restricted running period, rats were sacrificed by CO_2_ sedation followed by decapitation at ZT0, ZT6, ZT12, or ZT18, and the liver (left lateral lobe) and right soleus muscle were rapidly dissected, snap frozen in liquid nitrogen, and stored at −80 °C until further analysis. Sample size: at ZT0 (NR, n = 3; ALR, n = 6; DR, n = 6; LR, n = 6), at ZT6 (NR, n = 8; ALR, n = 6; DR, n = 3; LR, n = 5), at ZT12 (NR, n = 4; ALR, n = 7; DR, n = 6; LR, n = 6), and at ZT18 (NR, n = 7; ALR, n = 5; DR, n = 6; LR, n = 7). In a subgroup of rats, fat pads were also dissected and weighed.

##### 2.3.1.2 Experiment 2: running and eating restricted to light or dark phase

The experimental procedures were the same as in Experiment-1, except for the followings: after one week of acclimatization, rats were randomly assigned to one of the following three experimental groups in pairs instead of solitary (Fig. 4A): ad-libitum feeding and no access to running wheel (NR), access to the running wheel and food restricted to ZT13-23 in the dark period (DRDF); access to the running wheel and food restricted to ZT1-11 in the light period (LRLF). Animals were pair-housed in this experiment for reasons of experimental efficiency and based on the results of In het Panhuis et al. (2020). Sample size: at ZT0 (NR, n = 6; DRDF, n = 6; LRLF, n = 6), at ZT6 (NR, n = 6; DRDF, n = 6; LRLF, n = 6), at ZT12 (NR, n = 6; DRDF, n = 6; LRLF, n = 6), and at ZT18 (NR, n = 5; DRDF, n = 6; LRLF, n = 6). In Experiment 2, no subgroup of rats was available for weiging individual fat pads.

### 2.4 RNA isolation, cDNA synthesis, and RT-qPCR

RNA isolation, cDNA synthesis, and RT-qPCR were performed as described earlier (de Goede et al., 2018b). Primer sequences and housekeeping genes that were used for each tissue are listed in Supplemental Table S1.

### 2.5 Statistics

Normal distribution was confirmed using normal QQ plot. Data are presented as mean ± SEM. The RT-qPCR data were analyzed using the LightCycler conversion and LinReg software. GraphPad Prism 8 was used to perform statistical tests, and to visualize the data. Assessment of effects in experiments involving several conditions was performed using one- or two-way analysis of variance (ANOVA), with repeated measures where applicable, and followed by Tukey HSD *post hoc* tests to adjust for multiple comparisons when appropriate. Cosinor-based rhythmometry analyses were performed with CosinorPy (Moškon, 2020) using Python 3.8.5.

## 3 Results

### 3.1 Effects of time-restricted running (Experiment-1) on physiology

During the basal running period, ALR, DR and LR rats gradually increased their daily running distances in a similar manner (Fig. 1B). In addition, the 24 h running pattern was comparable between the three experimental groups, with the majority of running done during the dark (active) phase (Fig. 1B and C). LR rats adjusted within severaly days to the wheel availability restricted to the light (inactive) phase, and ran roughly 1.5 km/day (Fig. 1B–D). During the final days of running restricted to light phase, LR rats ran approximately 50% of the daily distance of the ALR and DR rats (Fig. 1B–D). LR rats ran the most during the first 4 h of their 10 h running period, but with a drastically reduced speed (m/h) compared to ALR and DR rats (Fig. 1D). ALR rats showed modest running activity at the beginning and end of the light phase, while DR rats displayed a sharper running peak than ALR rats during the early dark phase likely due to the lack of wheel access during the light period (Fig. 1D).

During the first days of unrestricted running, ALR, DR and LR rats had significantly lower daily caloric intake compared to NR rats, but these differences were no longer apparent by day 17 (Fig. 1E). During the restricted running period, daily food intake did not differ between the four experimental groups. However, when caloric intake was normalized to body weight, DR and ALR rats consumed relatively more calories than NR and LR rats (Fig. S1, left). Analysis of day- and nighttime food intake during the final week of the experiment revealed that LR rats consumed significantly more calories during the light phase than NR and DR rats (Fig. 1F). During the dark phase, both DR and ALR groups consumed significantly more calories than LR rats (Fig. 1F).

All experimental groups increased their body weight throughout the experiment (Fig. 1G). From day 3 onward, all three running groups gained significantly less body weight compared to the sedentary NR rats (Fig. 1G). LR rats weighed significantly more compared to DR rats from day 31 onwards and significantly more compared to ALR rats from day 38 onwards. Body weight gain after the start of time-restricted running was significantly higher in the LR rats than in DR and ALR groups, while it did not differ significantly from the NR rats (Fig. 1H). NR rats gained fat mass (Fig. 1I) and lost lean mass (Fig. 1K) throughout the experiment. In contrast, all runners displayed a significant decrease in fat mass, and an increase in lean mass compared to the NR group after the start of the experiment (Fig. 1I&K). Relative fat mass in LR rats was ∼50% higher than both ALR and DR groups after day 38 and this difference remained significant until the end of the experiment (Fig. 1I). Looking at fat gain since the start of time-restricted running, LR rats gained ∼1% more (as a percentage of body weight) fat than both ALR and DR groups after day 31 (Fig. 1J) and this difference grew larger.

Lean mass did not differ between running groups until day 45, where LR rats displayed 0.015 units (1.5%) lower lean mass/body weight ratio than DR while not differing from ALR (Fig. 1K). Body weight (Fig. 1G), relative fat (Fig. 1I), and lean mass (Fig. 1K) did not significantly differ between ALR and DR groups at any timepoint. At sacrifice, NR rats had the largest epididymal, subcutaneous, and perirenal fat depots, whereas LR rats had significantly more perirenal fat compared to both ALR and DR groups, as well as more epididymal fat compared to ALR rats (Figs. S2A–C).

**Fig. 2:**
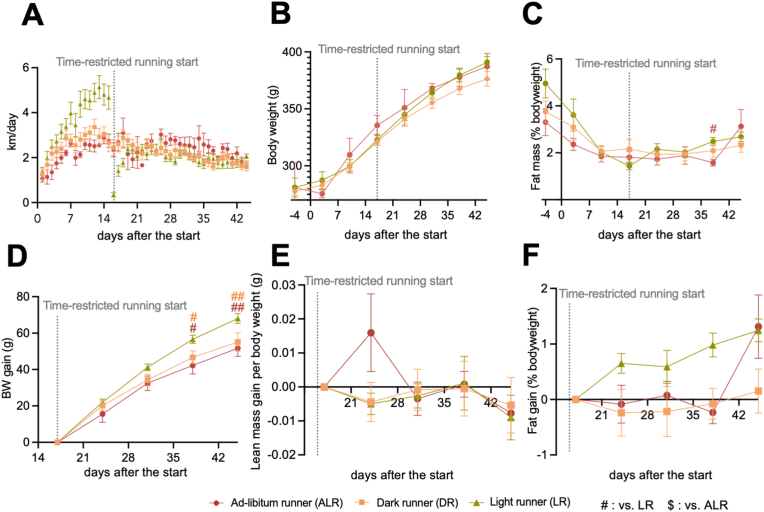
Light running is less efficient for preventing weight gain. **A:** Daily running distances of the selected animals from each group that ran approximately equal distances during the time-restricted running period (DR: n = 12, LR: n = 15, ALR: n = 4). **B:** Body weight development. **C:** Fat mass in percentage per body weight. **D:** Gain of body weight, **E:** Gain of lean mass per body weight. **F:** Gain of fat percentage **D-F:** From the start of the time-restricted period. The start of time-restricted running is represented by a grey dotted line. Data are presented as the mean ± SEM. Significant difference from light runner (LR) (#), or from ALR ($) compared to the groups of color code. # or $: *P* < 0.05, ## or $$: *P* < 0.01 by two-way ANOVA followed by Tukey HSD *post-hoc* test. (For interpretation of the references to color in this figure legend, the reader is referred to the Web version of this article.)

Within each running group, a noticeable variation in the daily running distances of individual animals was observed. Interestingly, every running group contained some rats that ran about the same daily distance during the time-restricted running period (i.e. 2,1 ± 0,6 km; Fig. 2A, Fig. S3, Table S2). No significant difference in body weight was apparent between the ALR, DR and LR groups when focusing on these “equal distance runners”, during either the basal or time-restricted period (Fig. 2B). Same goes for the fat percentage except for day 38 between ALR and LR (Fig. 2C). When focusing on the change after the start of time-restricted period, LR rats displayed a higher weight gain compared to DR rats, and ALR rats, resulting in 15 ± 2 g higher body weight in LR at the end of the experiment (Fig. 2D). However, changes in fat mass and lean mass did not show any significant differences among the three groups (Fig. 2E,F).

### 3.2 Effects of time-restricted running (Experiment-1) on clock gene rhythms

To assess the impact of wheel running restricted to the light or dark phase on peripheral clock function, we measured clock gene expression rhythms in liver and soleus muscle tissues, as this skeletal muscle is most sensitive to wheel running and detraining (Hyatt et al., 2019). In the liver, a significant *Group × Time* (interaction effect) was observed in *Dbp* and *Reverba*. Despite minor differences in *Dbp* and *Reverba* expression at some time points, no major significant group difference were found (Fig. S4A, Table S3). Cosinor analysis was performed to identify the acrophase, amplitude and 95% confidence interval of both acrophase and amplitude (Fig. S4B, Tables S4–S5). *Cry1, Per2,* and *Reverba* genes phase-advanced for 1,6 ± 0,2 h in LR compared to NR while only LR *Reverba* also differed from ALR and DR. This means that time-restricted running did not result in a major shift of the liver clock. Curiously, in DR, the *Per2* amplitude was reduced as compared to that in the LR and NR groups. The remaining genes showed no effect of light nor dark phase running on the amplitude.

**Fig. 3:**
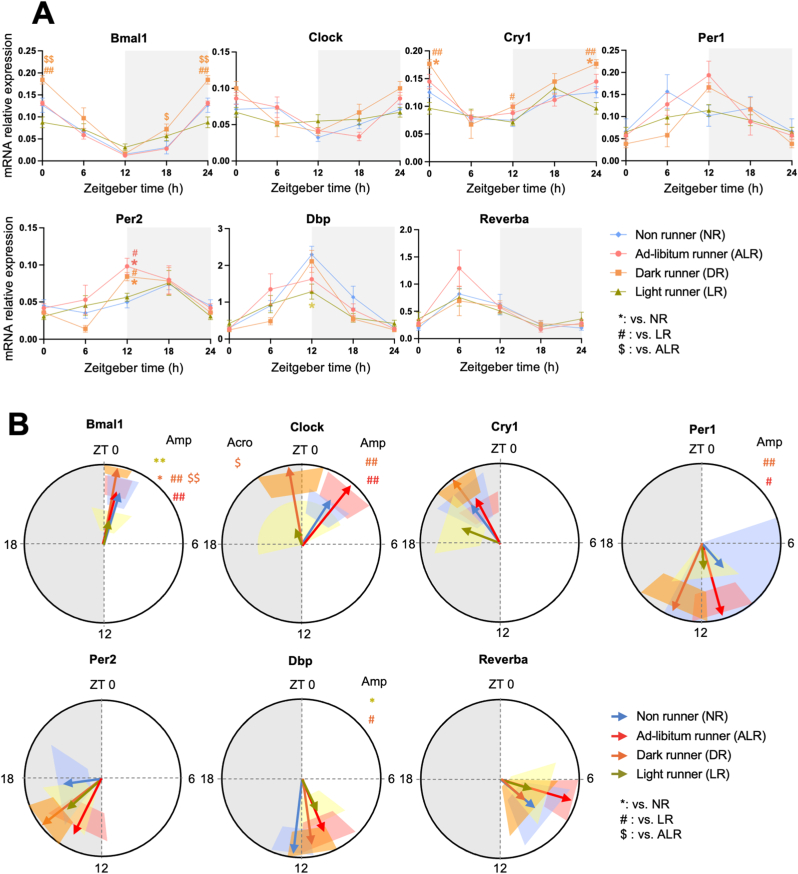
Four weeks of light phase running dampen, and dark phase running strengthen the expression profiles of clock genes in rat soleus muscle. **A:** mRNA relative expression analyzed by two-way ANOVA followed by Tukey HSD *post-hoc* test. **B:** Acrophase (indicated by the direction of arrows) with their amplitude (indicated by the length of arrows) of the clock (controlled) genes were analyzed by cosinor-based rhythmometry analysis using CosinorPy. CosinorPy adjusts the significance values using the false discovery rate (FDR) method (reported as *q*-values). Signs in the right top corner of each circular figure represent significant differences on amplitude. Grey shaded area represents the dark (inactive) phase. Coloured shaded areas (corresponding to the group colour code) in B represent 95% confidence interval. ZT = Zeitgeber time, h = hour (time). Amp: Amplitude. Acro: Acrophase. At ZT0 (NR, n = 3; ALR, n = 6; DR, n = 6; LR, n = 6), at ZT6 (NR, n = 8; ALR, n = 6; DR, n = 3; LR, n = 5), at ZT12 (NR, n = 4; ALR, n = 7; DR, n = 6; LR, n = 6), and at ZT18 (NR, n = 7; ALR, n = 5; DR, n = 6; LR, n = 7). Data are presented as the mean ± SEM. Significant difference from NR (∗), or from ALR ($), or from LR (#) compared to the groups of color code. ∗ or # or $: *P* < 0.05, ∗∗ or ## or $$: *P* < 0.01. (For interpretation of the references to color in this figure legend, the reader is referred to the Web version of this article.)

Analysis of soleus muscle revealed a significant *Group × Time Interaction* effect in half of the major clock genes that we tested (*i.e. Bmal1, Cry1, Per2*, and *Dbp* (Fig. 3A, Table S3)). Also, significant *Group* effects were observed. However, cosinor analysis revealed that no phase shifts were triggered by light or dark phase running except for a phase advance of *Clock* in DR compared with ALR (Fig. 3B–Table S4). Intriguingly, the greatest effect of time-restricted running was observed not on the diurnal phase, but on the diurnal amplitude of several clock genes in soleus muscle (Fig. 3B). The amplitude of daily *Bmal1, Clock, Per1* and *Dbp* gene expression was significantly dampened in LR rats compared to that in DR rats. LR rats clearly dampened *Bmal1* and *Dbp* expression even in comparison with NR rats. In contrast, in DR rats, *Bmal1* expression showed an enhanced amplitude compared to ALR, despite their running hours being quite comparable. Compared to NR rats, *Bmal1, Clock,* and *Per1* amplitudes were enhanced in DR and ALR groups, but not in LR rats.

**Fig. 4:**
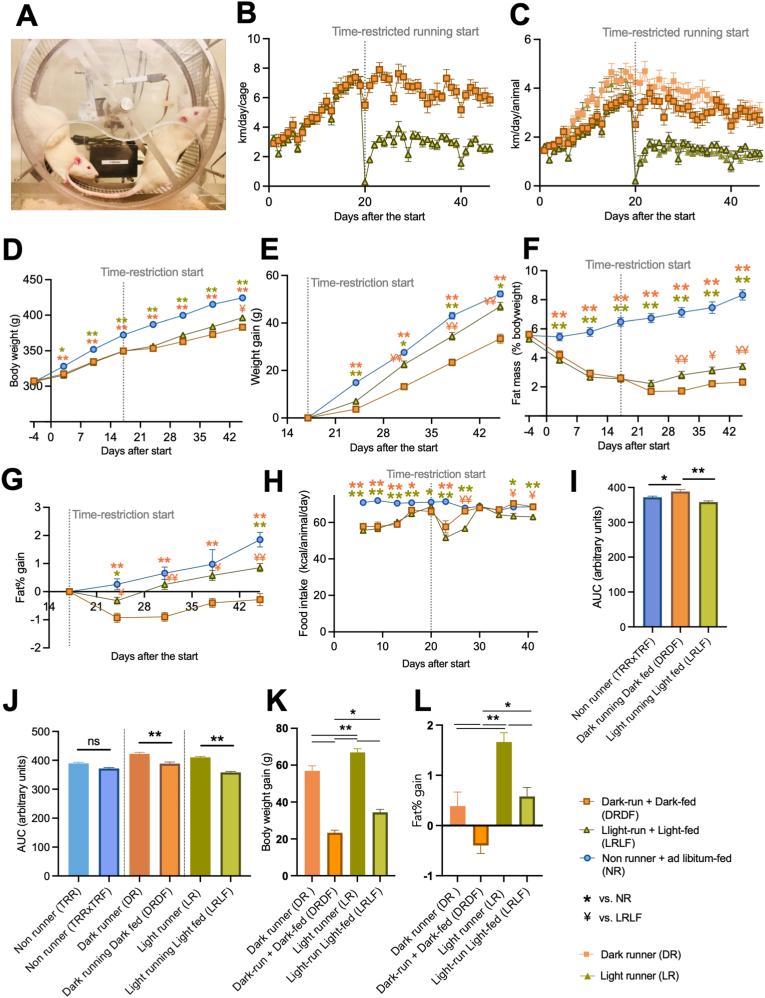
Combining time-restricted wheel running and time-restricted feeding (TRRTRF) in the light phase has less beneficial physical outcomes than combining running and feeding in the dark phase. **A:** A photo of 2 Wistar rats running together in a wheel. **B:** The average daily running distance of each group per cage (2 rats/cage) during the TRRTRF experiment. **C.** The average daily running per animal during Experiment-2 in comparison with the time-restricted running experiment (single-housed, animals from Experiment-1 (Fig. 1B)). **D:** Body weight development. **E:** Gain of body weight from the start of the time-restriction period. **F:** Fat mass in percentage per body weight. **G:** Gain of body fat from the start of the time-restricted running period. **H:** Daily food intake. **I:** Area under the curve of food intake per rat per 100 g body weight from the start of the time-restriction period till the final week of the experiment. **J:** Comparison between TRRxTRF (Experiment-2) and TRR alone (Experiment-1) for area under the curve of food intake per rat per 100 g body weight from the start of time-restriction period till the final week of the experiment. **K:** Comparison between TRRxTRF and TRR alone for total average body weight gain from the start of time-restriction period till the final week of the experiment per group. **L:** Comparison between TRRxTRF and TRR alone for total average fat gain from the start of time-restriction period till the final week of the experiment per group. Dark running dark fed group (DRDF, in orange with brown frame): n = 24, Light running light fed group (LRLF, in yellow green with olive frame): n = 24, Sedentary non runner (NR, blue with dark blue frame): n = 23. Data are presented as the mean ± SEM. Significant difference from NR (∗) or from LR (#) compared to the groups of color code. ∗ or #: P < 0.05, ∗∗ or ##: P < 0.01 by one-way or two-way ANOVA followed by Tukey HSD *post-hoc* test. (For interpretation of the references to color in this figure legend, the reader is referred to the Web version of this article.)

### 3.3 Effects of combined time-restricted running and feeding (experiment 2) on physiology

The next experiment was performed to examine if restricting both running and feeding time to the light phase could shift the muscle clock. To confirm that the change from solitary to pair-housing (Fig. 4A) did not affect their running behavior, changes in daily running distance were carefully observed throughout the experiment. As the results show, both the dark running dark fed group (DRDF) and the light running light fed group (LRLF) increased their daily running distances during the basal period in a very similar manner as in the DR and LR groups in Experiment 1 (Fig. 4B). When comparing to the time-restricted running data, the average running distance per animal was comparable in both DRDF and LRLF compared to the groups of the same time-restricted running phase (DR or LR) in Experiment 1 (Fig. 4C). In addition, we confirmed that the hourly running patterns between DRDF and DR, and between LRLF an LR were similar in the final week of the experiment despite that the peaks seemd a little more pronounced in the pair-housed cohorts (Fig. 1D and Fig. S5). All three groups, i.e. NR, DRDF and LRLF, showed an increase in body weight throughout the experiment. From day 3 onward, the NR rats gained more body weight than the two running groups (Fig. 4D). At the end of the experiment,

LRLF rats were 13 g heavier than those in the DRDF group. LRLF showed higher weight gain than DRDF from day 31 (Fig. 4E). Fat mass was also the highest in NR from day 3 onward, and LRLF showed higher fat mass compared to DRDF from day 31 (Fig. 4F). Fat gain became significantly higher in LRLF than DRDF from day 24 (Fig. 4G). Surprisingly, DRDF was the only condition that showed a continuous fat loss from the start of the time restriction. Throughout the time-restricted phase, LRLF rats ate generally less than NR and DRDF groups (Fig. 4H). When caloric intake was normalized with body weight, LRLF rats ate the least while DRDF rats ate the most during the time-restricted phase (Fig. 4I). Comparing the body weight corrected caloric intake between experiments 1 and 2, NRs between experiments did not differ, while both DRDF and LRLF ate significantly less compared to DR and LR (Fig. 4J). When comparing the body weight gain since the start of time restriction, DRDF was the lowest followed by LRLF, DR, and the heaviest was LR. When comparing the fat% gain since the start of restriction, DRDF showed the highest amount of fat loss, followed by DR, LRLF and the biggest gain in LR (Fig. 4L).

### 3.4 Effects of combined time-restricted running and feeding (experiment 2) on clock gene rhythms

To assess the impact of combined time-restricted running and time-restricted feeding on peripheral clock function, we measured clock gene expression rhythms in liver and soleus muscle tissues.

**Fig. 5:**
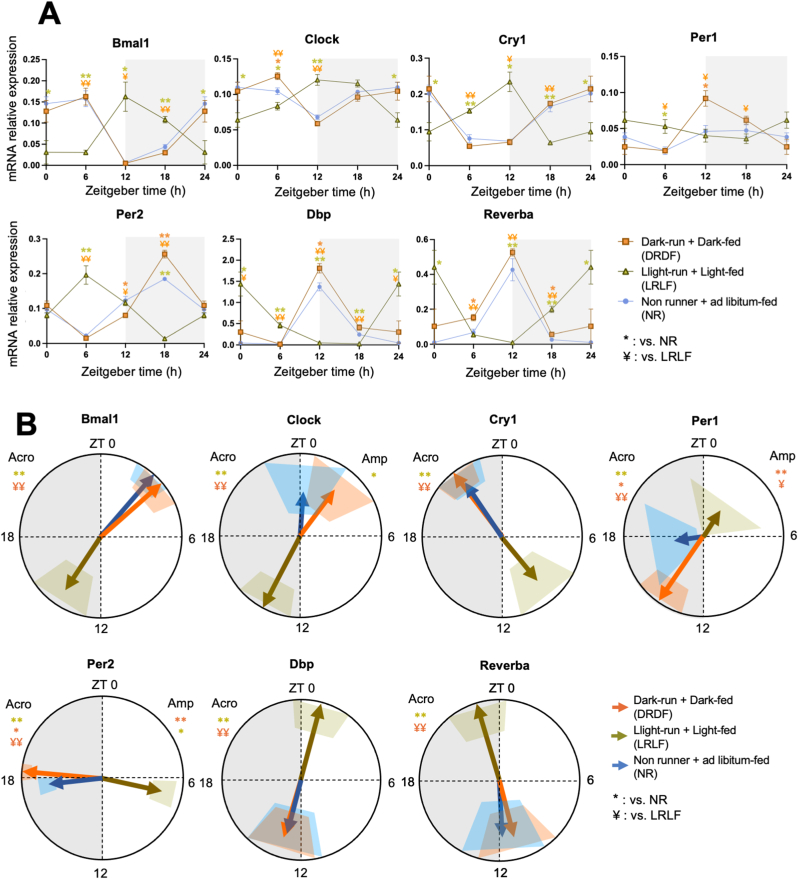
Four weeks of combined running and feeding during the light phase shifts and dampens the expression profiles of clock genes in rat liver. **A:** mRNA relative expression analyzed by two-way ANOVA followed by Tukey HSD *post-hoc* test. **B:** Acrophase (indicated by the direction of arrows) with their amplitude (indicated by the length of arrows) of the clock (controlled) genes were analyzed by cosinor-based rhythmometry analysis using CosinorPy. CosinorPy adjusts the significance values using the false discovery rate (FDR) method (reported as *q*-values). Signs in the right top corner of each circular figure represent significant differences in amplitude. Signs in the left top corner of each circular figure represent significant differences in acrophase. Grey shaded area represents the dark (inactive) phase. Coloured shaded areas (corresponding to the group colour code) in B represent 95% confidence interval. ZT = Zeitgeber time, h = hour (time). Amp: Amplitude. Acro: Acrophase. At ZT0 (NR, n = 6; DRDF, n = 6; LRLF, n = 6), at ZT6 (NR, n = 6; DRDF, n = 6; LRLF, n = 6), at ZT12 (NR, n = 6; DRDF, n = 6; LRLF, n = 6), and at ZT18 (NR, n = 5; DRDF, n = 6; LRLF, n = 6). Data are presented as the mean ± SEM. Significant difference from NR (∗), or from LRLF (#) compared to the groups of color code. ∗ or #: *P* < 0.05, ∗∗ or ##: *P* < 0.01. (For interpretation of the references to color in this figure legend, the reader is referred to the Web version of this article.)

In the liver, a significant *Group × Time* interaction effect was observed in all clock genes tested (Fig. 5A, Table S6). Cosinor analysis revealed that LRLF drastically shifted the acrophase of all the clock genes, by 12 h compared to DRDF and NR (Fig. 5B–Tables S7–S8). Comparing NR and DRDF, most genes did not show any phase difference, except for *Per1* and Per2. In DRDF, *Per1* displayed a 3 h phase advance compared to NR and *Per2* displayed a 1 h phase delay. For *Clock, Per1, and Per2,* the amplitude was also altered. LRLF significantly strengthened the amplitude of *Clock* and *Per2* compared to NR, while DRDF strengthened the amplitude of *Per1* compared to both NR and LRDF, and that of *Per2* compared to NR. The rest of the clock genes demonstrated no change in amplitude (Tables S7–S8).

**Fig. 6:**
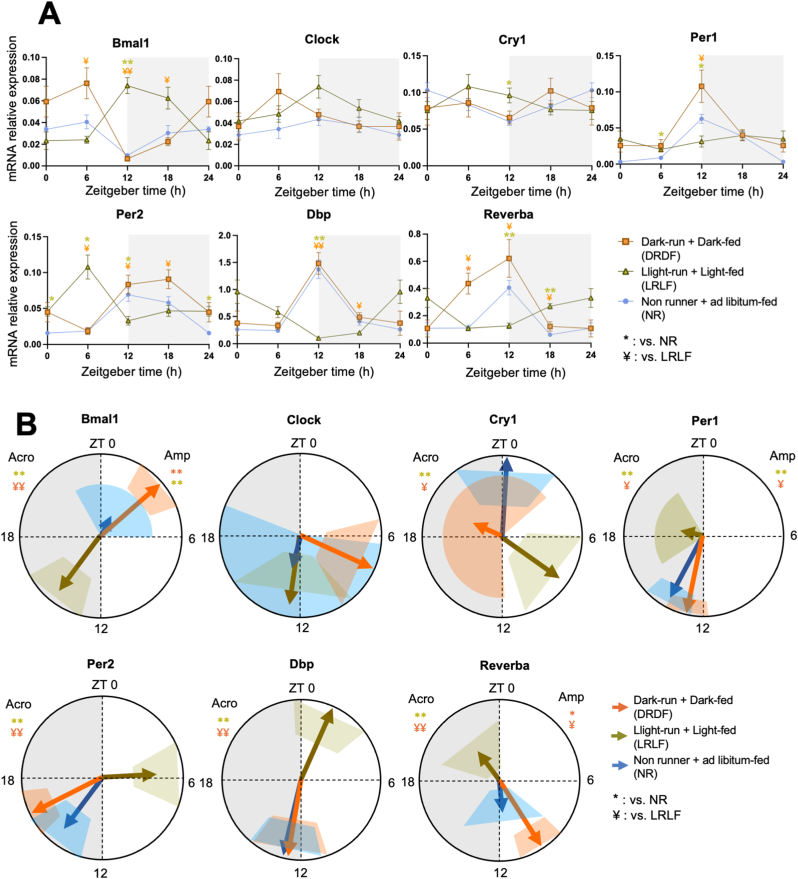
Four weeks of combined phase running and feeding during the light phase shifts and dampens the expression profiles of clock genes in rat soleus muscle. **A:** mRNA relative expression analyzed by two-way ANOVA followed by Tukey HSD *post-hoc* test. **B:** Acrophase (indicated by the direction of arrows) with their amplitude (indicated by the length of arrows) of the clock (controlled) genes were analyzed by cosinor-based rhythmometry analysis using CosinorPy. CosinorPy adjusts the significance values using the false discovery rate (FDR) method (reported as *q*-values). Signs in the right top corner of each circular figure represent significant differences in amplitude. Signs in the left top corner of each circular figure represent significant differences in acrophase. Grey shaded area represents the dark (inactive) phase. Coloured shaded areas (corresponding to the group colour code) in B represent 95% confidence interval. ZT = Zeitgeber time, h = hour (time). Amp: Amplitude. Acro: Acrophase. At ZT0 (NR, n = 6; DRDF, n = 6; LRLF, n = 6), at ZT6 (NR, n = 6; DRDF, n = 6; LRLF, n = 6), at ZT12 (NR, n = 6; DRDF, n = 6; LRLF, n = 6), and at ZT18 (NR, n = 5; DRDF, n = 6; LRLF, n = 6). Data are presented as the mean ± SEM. Significant difference from NR (∗), or from LRLF (¥) compared to the groups of color code. ∗ or ¥: *P* < 0.05, ∗∗ or ¥¥: *P* < 0.01. (For interpretation of the references to color in this figure legend, the reader is referred to the Web version of this article.)

In the Soleus muscle*,* a significant *Interaction* effect was observed in all 7 clock genes except for *Cry1* and *Clock* (Fig. 6A, Table S6). Cosinor analysis showed that LRLF shifted the acrophase of all clock genes by 11–12 h except for *Clock* and *Per1* (Fig. 6B–Tables S7–S8). *Per1* showed a phase shift of approx. 6 h. Between NR and DRDF, none of the clock genes showed any phase difference. When focusing on the amplitude, DRDF strengthened the amplitude of *Per1* and *Reverba* compared to LRLF, and *Per1*, *Reverba* and *Bmal1* compared to the NR group. Remarkably, LRLF also strengthened the amplitude of Bmal1 and Per1 compared to the NR group.

## 4 Discussion

Here, we demonstrated that only the combination of time-restricted feeding and time-restricted running can shift the muscle clock to stay aligned with the acrophase of the liver clock. All experimental groups with access to a running wheel showed reduced body weight gain and reduced adiposity compared to sedentary non runners, but the effectiveness differed between groups. For both body weight and fat gain prevention, combined dark-phase running and feeding (i.e. DRDF) was most effective while light-phase/daytime running (i.e. LR) was the least effective of all running groups with a ∼44 g (about 10% of rats’ BW before sacrifice) difference in weight gain and a ∼50% difference in fat gain between these groups. Also, when running comparable distances, wheel running mainly in the active phase (dark-phase running and ad-libitum running) significantly prevented weight gain compared to running in the inactive phase (light-phase running) (Fig. 2D).

Surprisingly, restricting running wheel activity to the light phase alone did not shift the muscle clock, even though these animals still ran almost 2 km per 12 h. Our results thus indicate that the reduced beneficial effects of running in the light phase on body composition are not due to a misaligned muscle clock. However, combining light phase running with light phase feeding did shift the muscle clock and re-aligned the muscle and liver clock, and this combination also provided more beneficial effects on body composition compared to light phase running alone. This suggests that alignment of peripheral clocks with the bevavioural rhythm may be more beneficial for metabolic health even if they are not aligned with the central clock (Smith et al., 2023). Most importantly, the fact that only the combination of feeding and exercise could shift the muscle clock (in contrast to the liver clock, that shifts with isolated time-restricted eating) shows that different organs are sensitive to different (combinations of) behavioral zeitgebers.

Despite the fact that light running took place during their habitual sleeping phase, Wistar rats chose to run voluntarily in the wheel during the light phase in agreement with the idea that wheel running is self-reinforcing (Mul, 2018; Mul et al., 2018). Light, *ad-libitum* and dark-phase running groups showed a pronounced peak in running wheel activity in the first half of their running phase. A sharper peak was observed in the dark running group than in the *ad-libitum* group, which is likely due to the limited access and the stimulus of locking and unlocking of the wheel.

When comparing rats with a comparable running distance in Experiment-1, both dark-phase running and *ad-libitum* running were significantly more efficient in preventing weight gain than light-phase running. This suggests that the beneficial metabolic effects of exercise are not only dependent on the amount of running, but also on time-of-day. Indeed, a previous study reported that early dark-phase exercise, but not exercise at the end of the dark phase, increased serum NEFA levels and triglyceride levels for up to 12 h after exercise in mice (Pendergrast et al., 2023). In contrast, another study reported that exercise in the late dark phase (ZT22), but not in the early dark phase (ZT13) reduced the fat mass by 19% in female mice (Schönke et al., 2023). Results from a study with mice on a high fat diet suggested that eating during the first part of the dark period with exercise in the late dark period is most efficient to prevent weight gain (Sasaki et al., 2014). Also in human epidemiology, the Netherlands epidemiology of obesity study (NEO study) reported that the timing of physical activity is associated with liver fat and other metabolic parameters (de Mutsert et al., 2013). It would have been interesting to compare rats running voluntarily in the first and second halves of the dark phase in our study, but only few rats showed a higher peak in the second half of the dark period; hence, the sample size was not sufficient for a separate analysis of these subgroups.

Surprisingly, we observed a reduction in the daily amplitude for some clock genes, but no major phase-shift of the Soleus muscle clock when rats could only run during the light period, with food available *ad libitum*. Most likely, this reduced amplitude is due to the abnormal timing of the physical activity in LR rats, since their feeding rhythm was only slightly affected and only minor effects on the liver clock were observed. In contrast, when only food intake is restricted to the light phase, the muscle clock became arhythmic (de Goede et al., 2018a). In this situation, home cage locomotor activity is divided fifty/fifty over the L/D cycle. Which indicates that exercise at the wrong time-of-day is not a strong enough stimulus to shift the muscle clock, but feeding at the wrong time-of-day seems to be since it makes the clock arhythmic. Therefore, the timing of feeding activity and exercise should be in line to maintain a rhythmic muscle clock. On the other hand, the liver clock is hardly affected by the timing of exercise.

Previous studies using *Per2::LUC* mice showed that voluntary wheel running (VWR) during either the early or late dark period delayed the phase of the liver clock by 2–4 h as compared to sedentary controls (Pendergast et al., 2014; Schroder et al., 2015), whereas VWR in the light period advanced the phase of the liver clock (Sasaki et al., 2016). In our study, light phase running phase-delayed the daily rhythms of *Cry1, Per2,* and *Reverba* in the liver clock by a maximum of 2 h. The difference between these results could derive from several factors such as a mice/rat species difference (Mavroudis et al., 2018), and/or also other variations in protocols and experimental settings. Obviously, in our protocol with only 4 sampling points over the 24 h L/D cycle, the temporal resolution is limited as compared to *Per2::LUC* studies. Indeed, guidelines for analysis of biological rhythms stated that simulations such as JTK_Cycle, as used in this study, are powerful but the phase estimation can be in accurate when using data sets collected less than every 4 h (Hughes et al., 2010, 2017; Hutchison et al., 2015). Therefore, it is challenging to compare our results with *Per2::LUC* studies, not only because of the species difference but also due to this difference in temporal resolution. So possibly, the fact that phase shift that were reported in *Per2::LUC* studies (Pendergast et al., 2014; Sasaki et al., 2016; Schroder et al., 2015) were not detected in our study is due to the lower sensitivity in our experimental setup. On the other hand, it is unlikely that the lack of phase shifts in the muscle clock in Experiment-1 is due to this lower temporal resolution, since phase shifts were detected with the same sampling protocol in Experiment-2.

Both treadmill and voluntary wheel running exercises during the light period have been reported to phase advance the molecular clock in the soleus muscle of *Per2::LUC* mice (Wolff and Esser, 2012). Interestingly, another study described that forced treadmill running enhanced the amplitude of only the clock gene *Per2* in the soleus muscle of C57BL/6 J mice (Casanova-Vallve et al., 2022). Our findings are most comparable to the latter as we observed no phase changes, but some changes in clock gene amplitude due to time-restricted running. Voluntary running has been reported to enhance the amplitude of *Per1, Per2* and *Nr1d1* expression in mice skeletal muscle, as compared to sedentary controls, which is also consistent with our findings (Yasumoto et al., 2015). Contrary to our rat study, it has been reported that in mouse models of time-restricted running, also the feeding rhythm shifted (Dalbram et al., 2019). Likely, this difference is due to the higher energy metabolism in mice versus rats (Fuller and Thyfault, 2021). At the same time, voluntary running in the dark period in obese high-fat diet-fed mice increased the abundance of skeletal muscle BMAL1 and CLOCK proteins (Dalbram et al., 2019), which seems to be in line with our findings of increased amplitudes of soleus *Bmal1* and *Clock* gene expression.

Time-restricted running during the light period shifted the phase of the liver clock with maximal 2–4hr and reduced the amplitude of the muscle molecular lock. This is a strong contrast to the 12hr shift in the liver clock and the arhythmic muscle clock when rats are subjected to time-restricted *feeding* during the light period (Bray et al., 2013; de Goede et al., 2018a; Oosterman et al., 2020; Opperhuizen et al., 2016; Reznick et al., 2013). Our previous work on time-restricted feeding showed that the timing of caloric intake also altered temporal dynamics of locomotor activity, with an almost equal distribution over the light/dark cycle with light-phase-restricted feeding (de Goede et al., 2019; De Goede et al., 2020; de Goede et al., 2018a, de Goede et al., 2018b; Oosterman et al., 2020). In Experiment-1, it was not possible to separate the relative contribution of physical activity and altered feeding behavior on the reduced amplitude of the muscle clock in LR rats. However, since the changes in the rhythm of caloric intake caused by time-restricted running were minor, voluntary wheel running is likely the strongest factor that contributed to the outcome of Experiment 1. At the same time, food availability/intake seems to be the strongest (or even only) non-SCN driver of the liver clock rhythm as suggested by previous findings. In contrast, for the soleus muscle clock, there may be multiple drivers. When combining daytime running with daytime eating, the majority of both liver and muscle clock genes showed a 12 h shift of their acrophase and yet their phases stayed aligned. Based on the fact that either time-restricted running alone or time-restricted feeding alone did not shift the muscle clock, our results indicate a differential sensitivity to the different (combinations of) Zeitgebers. The timing of food intake seems to determine the phase of the muscle clock, whereas the timing of exercise determines the amplitude of the muscle clock. In line, the latest multi-omics study on endurance training in Fischer 344 rats showed that 67% of the genes that responded to the training were tissue-specific (MoTrPAC Study Group, Lead Analysts, & MoTrPAC Study Group, 2024). Moreover, proteomics results suggested that liver and skeletal muscles displayed different regulations in protein abundance and post-translational modifications (Vieira et al., 2022). These findings could partially explain why differential organs have differential sensitivity towards various signals.

In addition, these differences in sensitivity to various combinations of Zeitgebers may be partly due to communication between skeletal muscle and the liver (Greco et al., 2021; Smith et al., 2023). During exercise, muscles use glucose at different rates depending on contraction intensity (Vieira et al., 2022). Simultaneously, the liver degrades glycogen to maintain blood glucose levels, which muscles use for ATP production. However, when ATP levels are high, the liver stores excess glucose as glycogen (Jensen et al., 2011). When exercise and eating occur at different times, glycogen supply and demand may not align. Additionally, there is a crosstalk also in immunometabolism. Exercise induces the production of interleukin-6 (IL-6), facilitating hepatic glucose output (Febbraio et al., 2004). Furthermore, exercise upregulates brain-derived neurotrophic factor (BDNF) mRNA and protein, as well as myokines that are released from muscle to the systemic circulation and penetrate the blood-brain barrier, eliciting an increase in cerebral BDNF levels (Pedersen and Febbraio, 2012). Moreover, elevated systemic IL-6 concentrations correlate with decreased food intake, indicating a potential role for circulating IL-6 in appetite regulation (Bay and Pedersen, 2020). This can partially explain the drop in food intake that we observed in the beginning of both Experiment-1 and 2. Despite the clear potential link between the differential tissue responses towards the timing of feeding and physical activity/exercise, how those Zeitgebers modulate the tissue specific clock gene landscapes is still not known. One previous finding suggests that the contraction of skeletal muscle increases the amplitude and phase-shifts of *Per2* via a calcium dependent pathway (Small et al., 2020). However, further studies will be required to dissect the exact mechanism of how differential (combinations of) pathways could shift peripheral tissue clocks.

## 5 Conclusion

This study demonstrates that synergy of time-restricted feeding and running is necessary to shift the muscle clock. It is the first study to explore the combined impact of time-restricted feeding and time-restricted running, while also assessing their individual effects. Our results indicate that both the timing of physical activity and food intake are important for an appropriately-timed muscle clock. Not only in rodents, but also in humans, the evidence to support the importance of coordinating the timing of physical activity and eating has been well documented. Time restricted eating in 24-h shift workers (firefighters) improved plasma cholesterol values, in patients with increased cardiovascular risk improved Hemoglobin A1C and diastolic blood pressure (Manoogian et al., 2022). In another study, 6 weeks of intermittent fasting (abstinence between 20:00pm to 12:00am, 16 h) improved body composition by decreasing the weight, BMI and fat mass (Domaszewski et al., 2020). Hopefully, our current findings will contribute to optimizing lifestyle strategies to prevent metabolic diseases, such as type 2 diabetes mellitus and obesity, caused by circadian disruption.

## Funding

A.S. was supported by Bioclock Consortium (by the NWA-ORC programme of the Dutch Research Council (NWO; project number 1292.19.077)). P.d.G was supported by a ZonMW TOP grant (#91214047).

## CRediT authorship contribution statement

**Ayano Shiba:** Writing – review & editing, Writing – original draft, Investigation, Formal analysis, Data curation. **Paul de Goede:** Writing – review & editing, Writing – original draft, Investigation, Formal analysis, Data curation, Conceptualization. **Roberta Tandari:** Investigation, Data curation. **Ewout Foppen:** Writing – review & editing, Supervision, Investigation. **Nikita L. Korpel:** Investigation, Formal analysis, Data curation. **Tom V. Coopmans:** Investigation. **Tom P. Hellings:** Investigation. **Merel W. Jansen:** Investigation, Formal analysis, Data curation. **Annelou Ruitenberg:** Investigation, Data curation. **Wayne I.G.R. Ritsema:** Investigation. **Chun-Xia Yi:** Writing – review & editing, Supervision. **Joram D. Mul:** Writing – review & editing, Supervision, Funding acquisition. **Dirk Jan Stenvers:** Writing – review & editing, Supervision, Funding acquisition. **Andries Kalsbeek:** Writing – review & editing, Validation, Supervision, Resources, Project administration, Methodology, Funding acquisition, Conceptualization.

## Declaration of competing interest

The authors declare the following financial interests/personal relationships which may be considered as potential competing interests:

Ayano Shiba reports financial support was provided by Bioclock Consortium. Paul de Goede reports financial support was provided by ZonMW. If there are other authors, they declare that they have no known competing financial interests or personal relationships that could have appeared to influence the work reported in this paper.

## Data Availability

Data will be made available on request.
