# Supplementary material for: Synergy between time-restricted feeding and time-restricted running is necessary to shift the muscle clock in male wistar rats
Source: Neurobiol Sleep Circadian Rhythms. 2024 Sep 19;17:100106. doi: 10.1016/j.nbscr.2024.100106 (PMC11462373; doi:10.1016/j.nbscr.2024.100106)
Supplement: Multimedia component 1 [file mmc1.pdf]

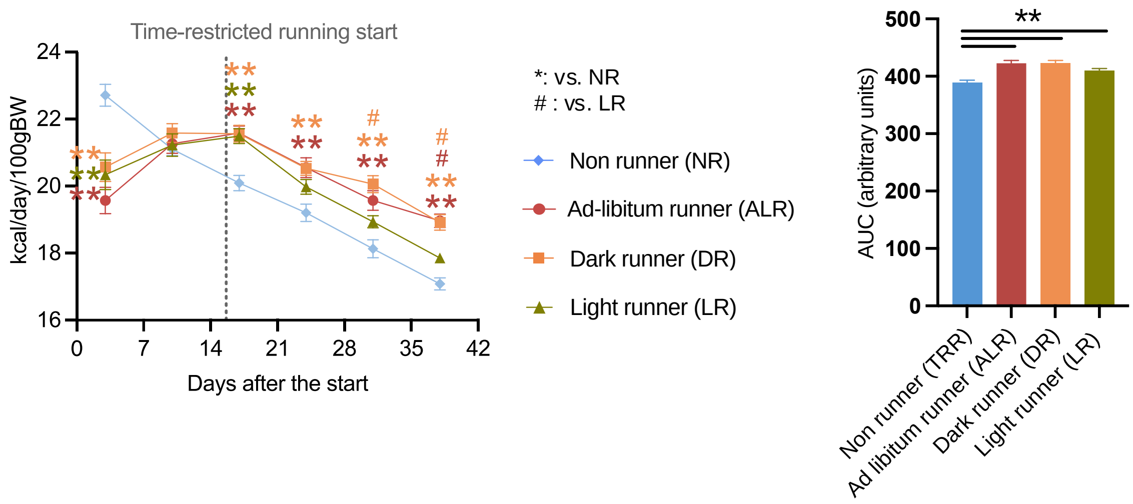

Supplemental figure 1.
Light runners and non-runners eat significantly less than Dark- or Ad-libitum runners during the time-restricted phase when food intake is expressed per 100g body weight. Left: Food intake per day per 100g body weight throughout the course of experiment. Right: Area under the curve of food intake per day per 100g body weight throughout the time restricted period. Sample size: non-runners (NR, in blue) n=36, Ad-libitum (ALR, in red) n=34, Dark (DR, in orange) n=36, Light (LR, in yellow) n=36. The start of time-restricted running is represented by a grey dotted line. Data are presented as the mean ± SEM. Significant difference from NR (*) or from LR (#) compared to the groups of color code. * or #: P < 0.05, ** or ##: P < 0.01 by one or two-way ANOVA followed by Tukey HSD *post-hoc* test.

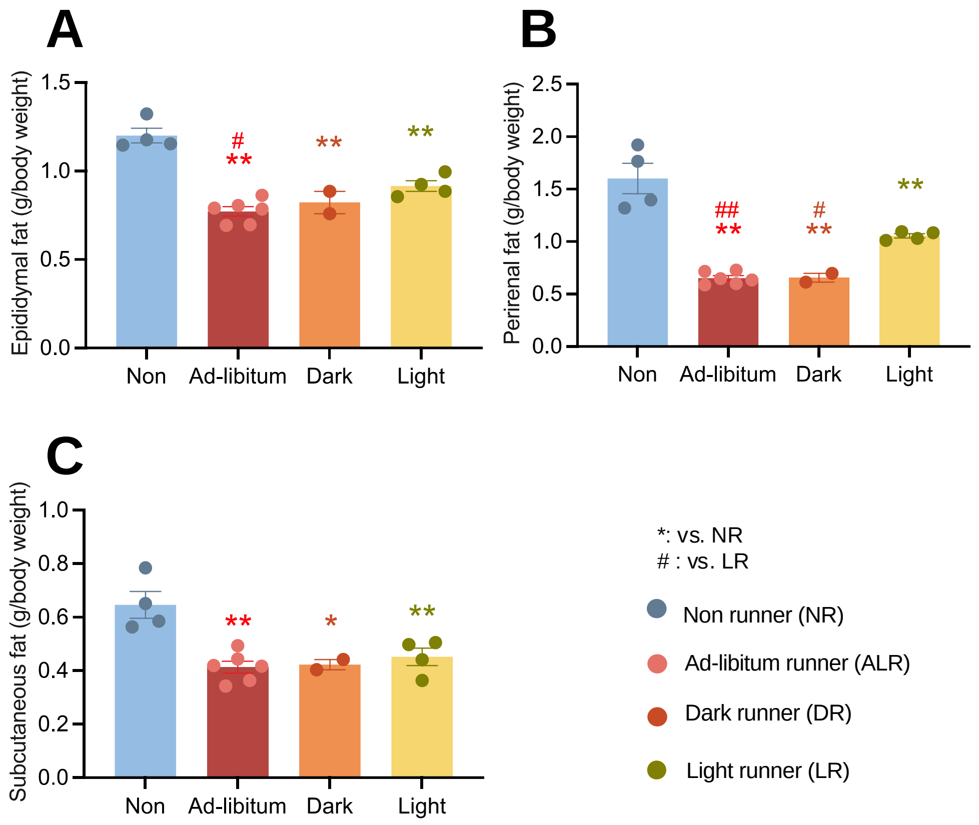

Supplemental figure 2.
Time-restricted running affects fat storage site-specifically. Epididymal fat (A), perirenal fat (B), and subcutaneous fat (C) were isolated after the sacrifice to investigate possible site-specific effect of time-restricted running. Sample size - non runner(NR) n=4, ad-libitum runner(ALR) n=6, dark runner(DR) n=2, light runner(LR) n=4. Data are presented as the mean ± SEM. Significant difference from NR (*) or from LR (#) compared to the groups of color code. * or #: $P < 0.05$, ** or ##: $P < 0.01$ by one-way ANOVA followed by Tukey HSD *post-hoc* test.

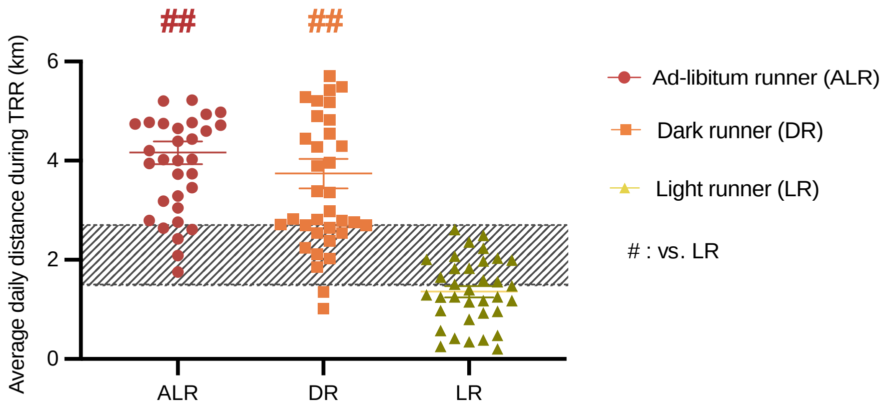

Supplemental figure 3.
The selection of animals that was made for the analysis shown in Fig. 2, based on their daily running distances during the time-restricted running period. The data of animals that ran 2,1±0,6 km (shaded area) were selected and extracted. Sample size - ad-libitum runner(ALR) n=5, dark runner(DR) n=12, light runner(LR) n=15. Data are presented as the mean ± SEM. Significant difference from LR (#) compared to the groups of color code. ##: P < 0.01 by one-way ANOVA followed by Tukey HSD *post-hoc* test.

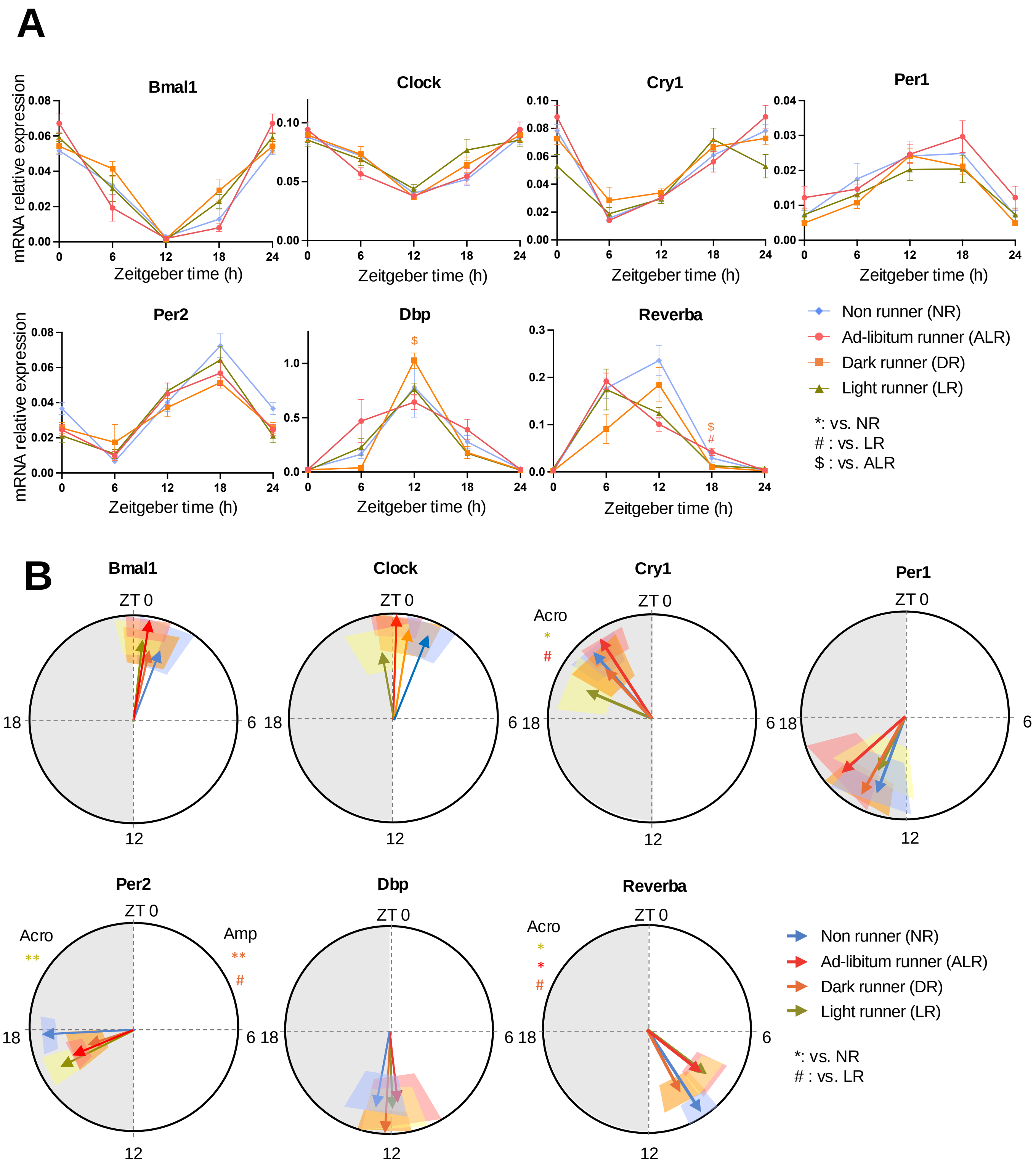

Supplemental figure 4.

Four weeks of time-restricted running partially shifts the daily expression profiles of clock (controlled) genes in rat liver. **A:** mRNA relative expression analyzed by two-way ANOVA followed by Tukey HSD *post-hoc* test. **B:** Acrophase (indicated by the direction of arrows) with their amplitude (indicated by the length of arrows) of the clock (controlled) genes were analysed by cosinor-based rhythmometry analysis using CosinorPy. CosinorPy adjusts the significance values using the false discovery rate (FDR) method (reported as *q*-values). Signs in the right top corner of each circular figure represent significant differences in amplitude. Signs in the left top corner of each circular figure represent significant differences in acrophase. Grey shaded area represents the dark (inactive) phase. Coloured shaded areas (corresponding to the group colour code) in B represent 95% confidence interval. ZT= Zeitgeber time, h= hour (time). Amp: Amplitude. Acro: Acrophase. At ZT0 (NR, n=4; ALR, n=6; DR, n=6; LR, n=6), at ZT6 (NR, n=8; ALR, n=6; DR, n=4; LR, n=6), at ZT12 (NR, n=4; ALR, n=6; DR, n=6; LR, n=6), and at ZT18 (NR, n=7; ALR, n=6; DR, n=6; LR, n=5). Data are presented as the mean ± SEM. Significant difference from NR (*), or from ALR ($), or from LR (#) compared to the groups of color code. * or # or $: $P < 0.05$, ** or ## or $$: $P < 0.01$.

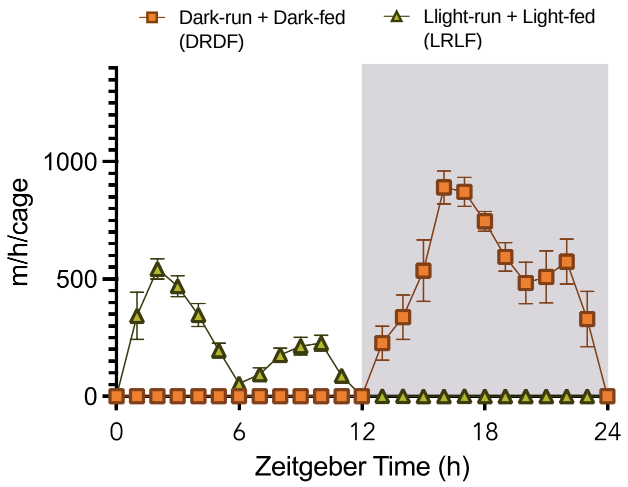

Supplemental figure 5.
Daily running pattern in DRDF and LRLF animals. The bigger peaks in running activity are observed during the first half of each running period. DRDF and LRLF data is per cage (2 rats per cage).

Supplemental Table S1
Summary of PCR primers

House-keeping gene	Symbol	Forward primer	Reverse primer	Used for
Beta2 macroglobulin	B2m	TGACCGTGATCTTTCTGGTGC	GCTTCCCATTCTCCGGTGG	liver
Beta-actin	Actb	ACAACCTTCTTGCAGCTCCTC	CTGACCCATACCCACCATCAC	muscle
Cyclophilin	Ppib	ATGTGGTCTTTGGGAAGGTG	GAAGGAATGGTTTGATGGGT	muscle
Glyceraldehyde-3-phosphate dehydrogenase	Gapdh	TGAACGGGAAGCTCACTGG	TCCACCACCCTGTTG CTGTA	liver
Hypoxanthine phosphoribosyltransferase 1	Hprt1	GCAGTACAGCCCCAAAATGG	AACAAAGTCTGGCCTGTATCCAA	muscle
TATA box-binding protein	Tbp	TTCGTGCCAGAAATGCTGAA	TGCACACCATTTTCCCAGAAC	liver
Sarcoplasmic/endoplasmic reticulum calcium ATPase 2	Serc2a	ATGGACGAGACGCTCAAGTT	GAAGCGGTTACTCCAGTATTGC	muscle
**Clock gene**	**Symbol**			
Brain and muscle arnt-like	Bmal1 (Arntl)	CCGATGACGAACTGAAACACCT	TGCAGTGTCCGAGGAAGATAGC	Both
circadian locomotor output cycles kaput	Clock	CGATCACAGCCCAACTCCTT	TTGCAGCTTGAGACATCGCT	Both
Cryptochrome Circadian Regulator 1	Cry1	AAGTCATCGTGCGCATTTCA	TCATCATGGTCGTCGGACAGA	Both
Period circadian regulator 1	Per1	CGCACTTCGGGAGCTCAAACTTC	GTCCATGGCACAGGGCTCACC	Both
Period circadian regulator 2	Per2	CACCCTGAAAAGAAAGTGCGA	CAACGCCAAGGAGCTCAAGT	Both
Rev-Erb alpha	Nr1d1	ACAGCTGACACCACCCAGATC	CATGGGCATAGGTGAAGATTTCT	Both
D site of the albumin promoter (albumin D-box) binding protein	Dbp	CCTTTGAACCTGATCCGGCT	TGCCTTCTTCATGATTGGCTG	Both

Supplemental Table S2

The variation in mean daily distances of rats that ran similar distances during TRR

	DR	LR	ALR
Geomean	2.1004	1.9587	2.2770
Standard deviation	0.5416	0.3370	0.3770
One way ANOVA	p value = 0.3936		

Geomean, standard deviation and p-values of One-way ANOVA tests of daily running distances during TRR of each running groups in Fig.2.

Supplemental Table S3

Summary of two-way ANOVA results of figure 3A and S4A

	**Liver**			**Soleus**		
**Genes**	**Time**	**Group**	**Interaction**	**Time**	**Group**	**Interaction**
Bmal1	**<0.0001**	0,2428	**0,0018**	**<0.0001**	**0,0001**	**<0.0001**
Clock	**<0.0001**	0,5594	**0,0445**	**<0.0001**	0,4033	**0,0008**
Per1	**<0.0001**	0,0779	0,9266	**<0.0001**	0,5741	**0,0453**
Per2	**0.0001**	0,1569	**0,0397**	**0.0017**	0,3848	0,1394
Cry1	**<0.0001**	0,2506	**0,0001**	**0.0051**	**0,0081**	**0,0001**
Reverb-α	**<0.0001**	0,0636	**0,0003**	**<0.0001**	0,7005	0,2301
DBP	**<0.0001**	0,5039	**0,0048**	**<0.0001**	0,2075	**0,0401**

Significant values **in bold letters.**

Supplemental Table S4

Summary of raw PCR clock gene values outputted by CosinorPy analysis for figure 3B and S4B

Liver						Muscle					
Parameter	Gene	ALR	DR	LR	NR	Parameter	Gene	ALR	DR	LR	NR
Amplitude	Bmal1	0,036	0,026	0,030	0,027	Amplitude	Bmal1	0,064	0,087	0,028	0,061
	Cry1	0,039	0,027	0,029	0,036		Cry1	0,035	0,053	0,029	0,033
	Clock	0,030	0,026	0,020	0,026		Clock	0,031	0,031	0,007	0,022
	Per1	0,010	0,011	0,008	0,010		Per1	0,069	0,067	0,025	0,032
	Per2	0,026	0,018	0,030	0,033		Per2	0,031	0,037	0,023	0,019
	Dbp	0,327	0,451	0,348	0,347		Dbp	0,745	0,866	0,465	0,972
	Reverba	0,095	0,097	0,103	0,135		Reverba	0,603	0,263	0,276	0,367
Peak (ZT)	Bmal1	0,577	0,769	0,432	1,345	Peak (ZT)	Bmal1	0,817	0,721	0,961	1,105
	Cry1	21,814	21,141	19,508	21,285		Cry1	22,006	21,526	19,219	21,429
	Clock	0,144	0,625	23,303	1,489		Clock	2,643	23,399	22,679	2,162
	Per1	15,183	13,934	13,790	13,309		Per1	10,955	13,502	11,580	9,369
	Per2	16,480	16,673	16,240	17,778		Per2	13,742	15,471	15,183	17,345
	Dbp	11,532	12,156	11,820	12,685		Dbp	10,523	11,483	10,330	12,492
	Reverba	8,500	10,130	8,460	9,800		Reverba	7,111	8,745	7,111	8,601

Supplemental Table S5
p-values corresponding to the t-test for cosinor analysis results of Fig. 3B & S4B
(Acrophase results are in the lower left part of the rectangle and Amplitude results in the upper right part of the rectangle.)
Significant values **in bold letters**.

Liver			amplitude			
			ALR	DR	LR	NR
acrophase	Bmal1	ALR		0.158950	0.314472	0.260571
		DR	0.815747		0.387729	0.783440
		LR	0.815747	0.815747		0.603679
		NR	0.726473	0.726473	0.608001	
	Cry1	ALR		0.306971	0.306971	0.795093
		DR	0.596109		0.830611	0.306971
		LR	**0.018981**	0.050333		0.439332
		NR	0.596109	0.837202	**0.046848**	
	Clock	ALR		0.555337	0.267940	0.555337
		DR	0.561223		0.367286	0.978108
		LR	0.481022	0.297283		0.367286
		NR	0.126966	0.297283	0.066474	
	Per1	ALR		0.906388	0.906388	0.967723
		DR	0.451754		0.647590	0.906388
		LR	0.578352	0.917036		0.906388
		NR	0.451754	0.893969	0.909591	
	Per2	ALR		0.183298	0.402176	0.150210
		DR	0.762546		**0.034238**	**0.004274**
		LR	0.762546	0.741934		0.402176
		NR	0.051973	0.209959	**0.008731**	
	Dbp	ALR		0.386681	0.989986	0.989986
		DR	0.769962		0.386681	0.480498
		LR	0.769962	0.769962		0.989986
		NR	0.769962	0.769962	0.769962	
	Reverba	ALR		0.890615	0.890615	0.088959
		DR	0.049622		0.890615	0.108990
		LR	0.939179	**0.049622**		0.172252
		NR	**0.049622**	0.745214	**0.049622**	

Muscle			amplitude			
			ALR	DR	LR	NR
acrophase	Bmal1	ALR		**4,30E-03**	**3,48E-05**	7,73E-01
		DR	0,912746		**3,47E-10**	**1,84E-02**
		LR	0,912746	0,912746		**5,26E-03**
		NR	0,912746	0,912746	0,912746	
	Cry1	ALR		0.250118	0.817753	0.817753
		DR	0.808404		0.143346	0.143346
		LR	0.086006	0.086006		0.817753
		NR	0.808404	0.896730	0.086006	
	Clock	ALR		0.928602	**0.003778**	0.294418
		DR	**0.039253**		**0.003778**	0.294418
		LR	0.260649	0.810857		0.067520
		NR	0.810857	0.092980	0.260649	
	Per1	ALR		0.919243	**0.014262**	0.225820
		DR	0.212571		**0.003193**	0.225820
		LR	0.762449	0.466280		0.919243
		NR	0.646795	0.269008	0.646795	
	Per2	ALR		0.694934	0.656408	0.428937
		DR	0.343971		0.428937	0.186792
		LR	0.453173	0.845863		0.694934
		NR	0.312773	0.343971	0.343971	
	Dbp	ALR		0.597247	0.238351	0.490765
		DR	0.486336		**0.021013**	0.597247
		LR	0.900378	0.465891		**0.021013**
		NR	0.175506	0.465891	0.175506	
	Reverba	ALR		0.059182	0.059182	0.199414
		DR	0.397893		0.889025	0.412653
		LR	0.998200	0.397893		0.430378
		NR	0.397893	0.998200	0.397893	

Supplemental Table S6
Summary of two-way ANOVA results of figure 5A and 6A

	Liver			Soleus		
**Genes**	**Time**	**Group**	**Interaction**	**Time**	**Group**	**Interaction**
Bmal1	**0,0032**	0,1933	**<0,0001**	0,1946	0,0851	**<0,0001**
Clock	**0,0085**	0,2685	**<0,0001**	**0,0443**	**0,044**	0,2421
Per1	**0,0141**	0,1241	**<0,0001**	**0,0001**	**0,0354**	**0,0003**
Per2	**<0,0001**	0,1627	**<0,0001**	**0,002**	**0,0047**	**<0,0001**
Cry1	**0,0027**	0,5852	**<0,0001**	0,5148	0,8898	0,1714
Reverb-α	**0,0019**	0,0688	**<0,0001**	**0,0004**	0,1007	**<0,0001**
DBP	**0,0003**	**0,0406**	**<0,0001**	**<0,0001**	0,732	**<0,0001**

Significant values **in bold letters.**

Supplemental Table S7
Summary of raw PCR clock gene values outputted by CosinorPy analysis for figure 5B and 6B

Liver					Muscle				
Parameter	Gene	NR	DR	LR	Parameter	Gene	NR	DR	LR
Amplitude	Bmal1	0,088	0,088	0,071	Amplitude	Bmal1	0,012	0,036	0,031
	Cry1	0,082	0,098	0,071		Cry1	0,021	0,009	0,019
	Clock	0,019	0,025	0,034		Clock	0,008	0,018	0,015
	Per1	0,015	0,038	0,015		Per1	0,033	0,037	0,010
	Per2	0,082	0,121	0,093		Per2	0,033	0,040	0,029
	Dbp	0,601	0,662	0,847		Dbp	0,501	0,485	0,487
	Reverba	0,185	0,188	0,251		Reverba	0,127	0,291	0,133
Peak (ZT)	Bmal1	2,739	3,267	14,222	Peak (ZT)	Bmal1	2,018	3,219	14,510
	Cry1	21,766	21,526	9,417		Cry1	0,192	19,508	8,360
	Clock	0,240	2,450	13,886		Clock	12,973	7,591	12,589
	Per1	17,297	14,270	2,306		Per1	13,837	12,781	18,835
	Per2	17,441	18,258	6,823		Per2	14,511	16,240	5,814
	Dbp	12,925	13,117	0,961		Dbp	12,829	12,637	1,586
	Reverba	11,772	11,003	22,871		Reverba	11,580	9,802	21,622

Supplemental Table S8
p-values corresponding to the t-test for cosinor analysis results of Fig. 5B & 6B
(Acrophase results are in the lower left part of the rectangle and Amplitude results in the upper right part of the rectangle.)
Significant values **in bold letters**.

Liver			amplitude		
			NR	DRDF	LRLF
acrophase	Bmal1	NR		0,966261	0,553684
		DRDF	4,37E-01		0,553684
		LRLF	**1,51E-40**	**6,03E-31**	
	Cry1	NR		0,544332	0,544332
		DRDF	7,61E-01		0,544332
		LRLF	**1,60E-34**	**1,68E-32**	
	Clock	NR		0,437298	**0,044329**
		DRDF	1,30E-01		0,377658
		LRLF	**1,46E-19**	**1,43E-22**	
	Per1	NR		**0,009562**	0,979326
		DRDF	**2,55E-02**		**0,012267**
		LRLF	**2,48E-06**	**2,03E-11**	
	Per2	NR		**0,001787**	0,339215
		DRDF	**2,44E-02**		**0,047273**
		LRLF	**8,2E-132**	**8,9E-158**	
	Dbp	NR		0,717029	0,330969
		DRDF	8,61E-01		0,544952
		LRLF	**3,05E-35**	**2,42E-24**	
	Reverba	NR		0,950981	0,483816
		DRDF	5,64E-01		0,483816
		LRLF	**4,66E-26**	**4,06E-21**	

Muscle			amplitude		
			NR	DRDF	LRLF
acrophase	Bmal1	NR		**0,00369**	**0,002909**
		DRDF	5,63E-01		0,577303
		LRLF	**4,90E-13**	**4,73E-26**	
	Cry1	NR		0,772484	0,788457
		DRDF	0,284291		0,772484
		LRLF	**0,000016**	**0,023582**	
	Clock	NR		0,324167	0,324167
		DRDF	0,189404		0,773199
		LRLF	0,883159	0,06088	
	Per1	NR		0,678361	**0,002385**
		DRDF	0,402638		**0,0207**
		LRLF	**0,008787**	**0,046844**	
	Per2	NR		0,608946	0,632664
		DRDF	6,10E-02		0,608946
		LRLF	**2,56E-17**	**5,90E-19**	
	Dbp	NR		0,985842	0,985842
		DRDF	8,68E-01		0,985842
		LRLF	**7,48E-25**	**1,57E-15**	
	Reverba	NR		**0,013269**	0,879316
		DRDF	2,83E-01		**0,013269**
		LRLF	**2,87E-12**	**7,40E-16**	
